# Symptoms of Myasthenia Gravis Obscured by Old Age and Unilateral Presentation

**DOI:** 10.7759/cureus.44737

**Published:** 2023-09-05

**Authors:** Celeste Gracey, Ricardo Balladares

**Affiliations:** 1 Internal Medicine, Campbell University School of Osteopathic Medicine, Lillington, USA; 2 Internal Medicine, WakeMed, Raleigh, USA

**Keywords:** geriatric medicine, neuromuscular junction disorders, geriatric neurology, bulbar palsy, blepharoptosis, elderly, unilateral, case report, myasthenia gravis

## Abstract

Myasthenia gravis (MG) is a neuromuscular junction disorder involving autoantibodies affecting the postsynaptic muscle membrane. We report an 81-year-old man who presented to the emergency department with three days of left facial droop, who later developed worsening bilateral ptosis, cervical weakness, dysphagia, and dysarthria following an assessment for Bell’s palsy. Ultimately, he was diagnosed with MG. This patient’s presentation was atypical and challenging. Specifically, the patient had droopy eyelids from a redundancy of skin and an anatomical neck droop, non-specific findings in older adults, which obscured the development of bilateral ptosis and cervical weakness, a classic sign of bulbar disease. The patient also presented with unilateral facial weakness, a rare finding in MG and concerning stroke in the elderly population. Our aim is to discuss the challenges of identifying MG in older populations and to discuss pharmacological challenges in assessing elderly patients with suspected bulbar palsies.

## Introduction

Myasthenia gravis (MG) is a rare but well-described autoimmune disease in which antibodies are directed at the nicotinic acetylcholine receptors (AChR) on the post-synaptic side of the neuromuscular junction. These autoantibodies often lead to the destruction and inactivation of AChR or associated proteins. The result is decreased neuromuscular transmission which gives the clinical findings of fluctuating muscle weakness. Symptoms typically begin with bilateral ptosis and diplopia, followed by involvement of non-ocular muscles [1,2]. These symptoms are often obscured in elderly populations due to pre-existing droopy eyelids and dysarthria. The disease incidence is also thought to be increasing in populations above 50 with rates remaining stable among the younger populations [3]. MG can occur at any age but most often presents in young women and elderly men. About 10% of MG patients have thymomas, causing a paraneoplastic MG that often resolves with thymectomy [4]. Younger patients tend to have thymic hyperplasia and older patients have thymic atrophy [4,5].

Symptoms are typically the most severe toward the end of the day when acetylcholine supplies are the lowest. Weakness improves with rest. Single-fiber EMG and nerve conduction testing demonstrate the fatigability of muscles and confirm the diagnosis. A recording electrode is placed on the endplate region of a muscle then the muscle is repetitively stimulated. In MG patients, the signals reduce in amplitude with repeated stimulations. A decrement of 10% is considered abnormal or diagnostic [6,7]. Pharmacological testing can be performed using a therapeutic trial of the anti-acetylcholinesterase (pyridostigmine). There are several antibody tests that can be helpful in diagnosis. The AChR antibody is found in about 89% of patients [7]. Additional findings include anti-MuSK (muscle-specific kinase) and anti-LRP4 (low-density lipoprotein receptor-related protein 4) antibodies [8].

A myasthenic crisis occurs when a worsening of symptoms causes life-threatening neuromuscular respiratory failure that may require intubation and mechanical ventilation. Stressors including infections, surgeries, and medications are associated with these crises, although they can happen spontaneously [9].

## Case presentation

**Figure 1:**
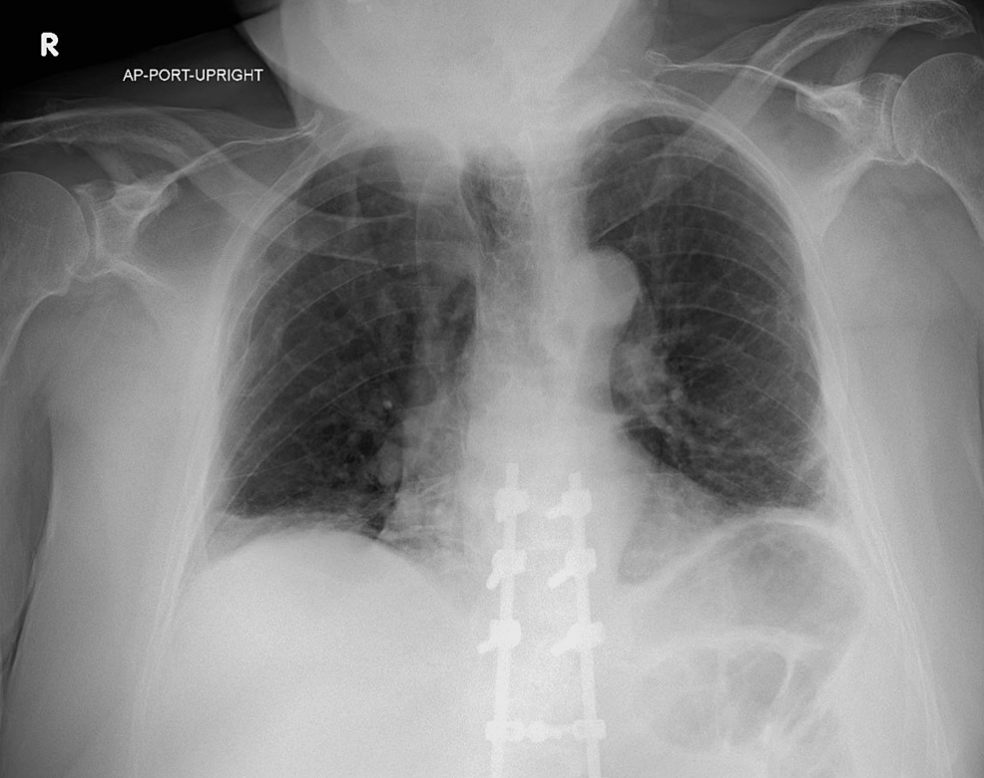
CXR on admission CXR on admission showed predominantly linear bibasilar opacities, low lung volumes, and subtle consolidations. The enlargement of the cardiopericardial silhouette was similar to prior imaging. It also shows partially imaged thoracolumbar posterior spinal fusion hardware and multiple remote bilateral rib fractures, both from prior motor vehicle collisions. CXR, chest x-ray

An 81-year-old man with a history of aortic stenosis with moderate cardiomegaly first noted on CT five years prior, traumatic motor vehicle collision requiring numerous spine fusions in the thoracolumbar region, obesity, and prostate cancer with radiation seven years prior presented with left facial weakness, dysarthria, dysphagia, and shortness of breath lasting one day. A day prior, he was seen in the emergency department with three days of facial weakness following the onset of a flu-like illness. The patient declined CT and MRI, because of issues laying flat. He was given valacyclovir and prednisone for presumed Bell’s palsy; however, he was unable to take the medications that night because of worsening dysphagia and returned to the emergency department the following day. On admission, he complained of a cough but denied diplopia, weight changes, chest pain, or fever. His oxygen saturation was 97% on room air, blood pressure was 181/74, pulse was 94, temperature was 97.8°C, respirations of 20, and a BMI of 32. On physical exam, the patient was unable to elevate either eyebrow. His family initially believed that was his baseline. His ptosis was only evident on the left, and he had difficulty closing the left eye. Both upper eyelids appeared to have a redundancy of skin causing mild baseline eyelid droop. He had a left-sided facial droop that produced an asymmetric smile. The strength in his bilateral extremities and neck was intact, but his neck drooped. The patient later explained that since the motor vehicle collision, his neck maintained a flexed position when he laid supine. Labs were significant only for elevated serial high-sensitivity troponins of 35 pg/mL and 42 pg/mL (reference range 0-18 pg/mL). An electrocardiogram revealed sinus rhythm with first-degree atrioventricular block. A chest x-ray (CXR) showed linear bibasilar opacities, subtle consolidations, and an enlargement of the pericardial silhouette (Figure 1). The patient was unable to complete a CT or MRI because of shortness of breath when lying flat. His echocardiogram was abnormal with hypokinesis and echodensities on the aortic valve and an estimated ejection fraction of 50-55% (Video 1).

**Video 1:** Echocardiogram with contrast Technically difficult echocardiogram shows slightly decreased left ventricular systolic function. The ejection fraction is estimated at 50-55%. There is global hypokinesis of the left ventricle with minor regional variation, including severe apical hypokinesis. There is severe sclerosis and moderate calcification of the aortic valve cusps and moderate aortic stenosis. There is a trivial amount of aortic regurgitation. Some mobile echodensities noted in one view of the aortic valve could represent degenerative changes.

Given the CXR findings and the fact that his breathing worsened in the supine position, a dose of IV furosemide was given, but it failed to improve the shortness of breath. We determined the opacities were likely related to aspiration of oral secretions. The initial clinical impression by a cardiologist attributed his inability to lay flat to anxiety. A speech and language pathologist found severe oropharyngeal dysphagia and severe flaccid dysarthria and recommended he take nothing by mouth. A nasogastric tube was placed for nutrition and medication, including valacyclovir and prednisone for Bell’s palsy.

For multiple nights while in the hospital, the patient developed respiratory distress with tachypnea, hypertension, and oxygen desaturation to the low 90s. The episodes would improve with 2-4 L of oxygen by nasal cannula. By morning they would resolve and the oxygen would be discontinued. Initially, it was thought that these episodes were brought on by anxiety, and the patient was treated with lorazepam as needed. His facial weakness continued to worsen with the development of bilateral ptosis, greater on the left. Diplopia also became evident through the finger-to-nose test, which he failed inconsistently. His dysarthria worsened during his stay, progressing to communication through writing only. The patient developed weakness in his shoulders and neck that eventually became noticeable even with his baseline neck droop. On his fourth day of admission, the patient received a brain MRI with the use of general anesthesia that did not reveal any acute ischemic infarcts or lesions that could explain his symptoms.

A neurologist was consulted, and they were impressed by the cervical weakness that he developed over his hospital course. The patient was given a trial dose of pyridostigmine, which resulted in an improvement in his symptoms. Nerve conduction testing using the median and accessory nerves showed a decremental pattern in conduction with a 14% decrement, which was consistent with neuromuscular junction disease. In addition, his acetylcholine receptor antibodies were 4.24 nmol/L (positive >0.40 nmol/L), confirming a diagnosis of MG. A computer tomography chest with contrast was negative for thymoma.

Outcomes

A day prior to the patient’s initial treatment for Bell’s palsy, he had sought medical care for a viral illness, which can often trigger crises in MG patients. Given his cervical weakness and new onset nighttime oxygen desaturation that required a high-flow nasal cannula, we decided to treat his presentation as a myasthenia crisis. He received five plasmapheresis treatments as an inpatient. His pyridostigmine dosing was increased to 180 mg oral CR twice daily. His only symptom at the time of discharge was a mild right-sided ptosis.

## Discussion

Even though populations over the age of 50 present unique challenges for identifying MG, incidences are growing in this age group in many parts of the world [3,10]. While death does occur, often alongside comorbid conditions, most elderly patients respond well to therapy and have a good prognosis [10,11].

Key traits of MG presentation such as ptosis, diplopia, dysarthria, and facial muscle weakness that are readily observed in youthful faces are often obscured by preexisting features of elderly patients [3]. Shortly before discharge, our patient admitted that he had been struggling with nighttime ptosis for about a month and had been reclining in his chair to an almost supine position to watch television at night. It can be helpful to get collateral information from family members by asking for specifics about changes in their facial features and changes to speech in the weeks leading up to their presentation. On initial presentation, dysarthria and dysphagia typically occur alongside other symptoms, but they can occur as the sole symptom in MG [11]. Our patient's initial dysarthria was identified by his partner. While collateral information can be helpful in separating new neurologic symptoms from a patient’s baseline presentation, the changes might not always be apparent to family members either. When inquiring about forehead paralysis, the patient’s partner thought his eyebrows could not elevate even at baseline.

Diagnosis can also be challenging in older adults because of the high incidence of cerebrovascular disease [3]. Given our patient’s unilateral facial droop, dysphagia, and dysarthria, an acute stroke was high on the differential. Our patient’s brain MRI was negative for stroke, but we could not completely rule out a stroke. It is estimated that 6.8% of stroke consultations are for diffusion-weighted-image-negative ischemic strokes [12]. Rarely MG can mimic stroke symptoms [13]. Our patient was found to have elevated blood pressure, evidence of prior cardiac events by EKG, and hypokinesis of his heart, all indicating a possible cause of stroke. Even after our patient’s MRI was negative for acute events, an MRI-negative stroke remained high on our differential until he responded to a trial of pyridostigmine.

In patients with MG who are not yet diagnosed, treatment in an acute hospital setting can be complicated due to an iatrogenic risk of worsening MG symptoms from common pharmacological therapies. This patient initially received prednisone and valacyclovir for what was thought to be Bell’s palsy. Although prednisone is used for the treatment of MG, it may also cause transient worsening of symptoms. When he responded to pyridostigmine, we initially held his prednisone. It was then restarted to treat the MG crisis. Many of the medications associated with worsening MG are common pharmacological therapies such as multiple classes of antibiotics and drugs commonly used in the older adult population such as beta blockers and statins [14]. With his abnormal echocardiogram and hypertension, our patient was likely a candidate for some of these therapies, and he was referred to outpatient cardiology. It is important to remember that although these medications are associated with worsening myasthenia symptoms, this does not mean that they cannot be used in a patient with MG, but their benefits and risks should be taken into consideration.

Unilateral presentation of MG is rare but has been documented on a few occasions [15,16]. When our patient initially presented to the emergency department, he was treated for Bell’s palsy. Bell’s palsy is facial paralysis in the distribution of the seventh cranial nerve causing facial weakness and is the cause of about half of the peripheral facial nerve palsies [17]. A key feature of Bell’s palsy that distinguishes it from stroke is that Bell’s palsy involves the forehead, which was the case in our patient [18]. Like in the MG flare of our patient, Bell’s palsy is associated with viral illnesses. However, Bell’s palsy diagnosis could not explain the severe dysphagia and respiratory crises that our patient was experiencing.

## Conclusions

This case demonstrated some of the many difficulties in identifying new onset MG in older patients. Specifically, our patient had a baseline neck droop and droopy eyelids from a redundancy of skin, common benign findings in older patients, which obscured otherwise classic signs of bulbar disease. When examining older patients for dysarthria or facial droop, it can be helpful to establish a baseline with either family or by using recent images. Identifying MG in older patients can also be difficult, because of high incidences of cerebrovascular incidents in this population. While assessing patients for possible stroke, underlying causes of stroke are important; this case is a good reminder to keep a broad differential until a singular diagnosis is clear. Once MG becomes a likely differential, assessing hospital and home medications is an important step in screening for possible triggers. This patient’s unilateral presentation and prior initiation of treatment for Bell’s palsy were also unusual; however, if a patient presents with dysphagia and dysarthria, suspicion of Bell’s palsy should be low.
